# Supplementary material for: Functional characterization of the 12p12.1 renal cancer-susceptibility locus implicates BHLHE41
Source: Nat Commun. 2016 Jul 7;7:12098. doi: 10.1038/ncomms12098 (PMC4941055; doi:10.1038/ncomms12098)
Supplement: Supplementary Information — Supplementary Figures 1-13, Supplementary Tables 1-3 and Supplementary Reference [file ncomms12098-s1.pdf]

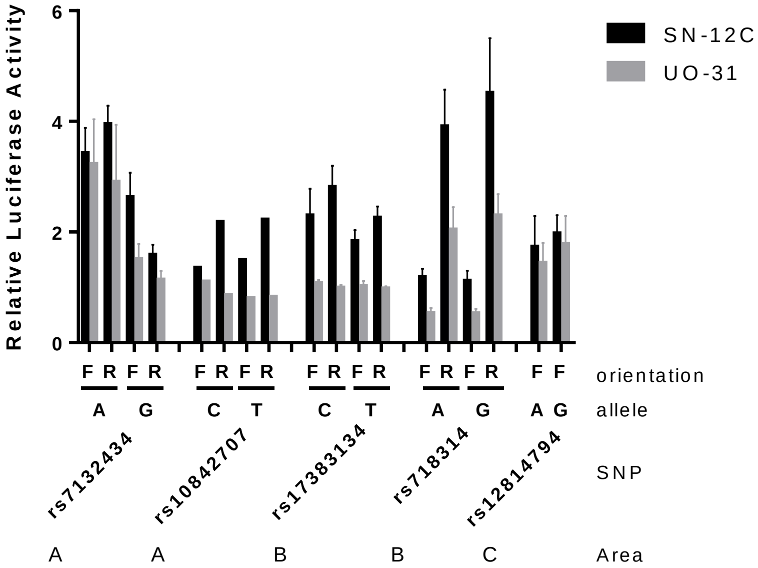

Supplementary Figure 1. Enhancer luciferase assay for 5 SNPs associated with RCC and localized in potential regulatory regions according to the ENCODE data tested in SN12C (dark grey) and UO31(light grey) renal cancer cell lines. DNA fragments containing the indicated allele of each of 5 SNPs were cloned in a forward (F) or reverse (R) orientation upstream of a minimal promoter that drives luciferase expression. A-C refers to the areas in Figure 1. The average relative luciferase activity of 3 independent experiments, performed in triplicate, is plotted; error bars represent the standard deviation.

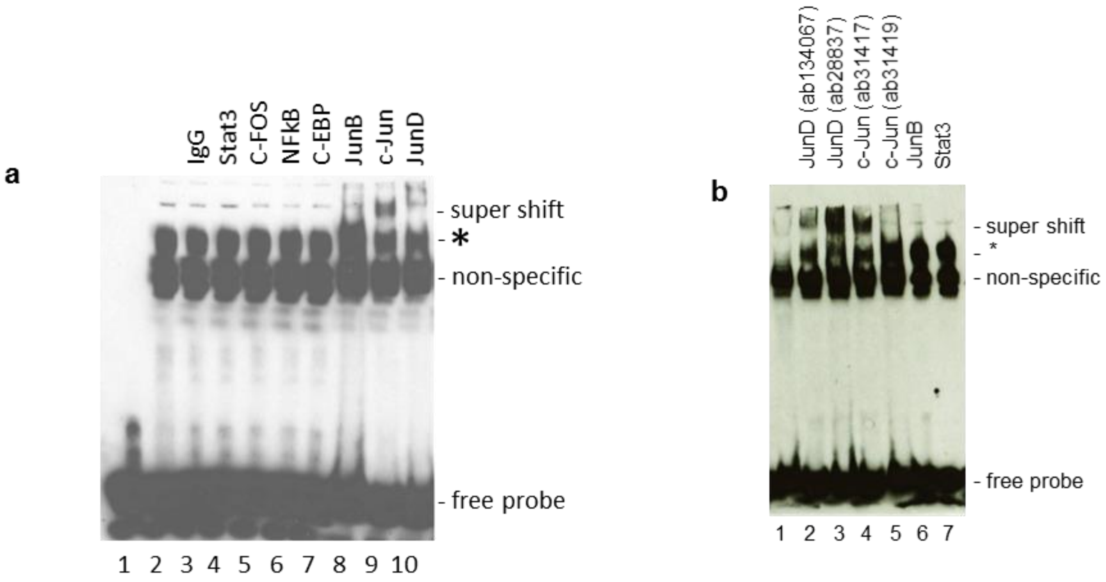

Supplementary Figure 2. EMSA of probe containing the risk allele (A) of rs7132434 with nuclear extract from UO-31 cells. (**a**) Supershift with indicated antibody. Lane 1 contains no extract. Lane 2 contains extract, but no antibody. (**b**) Supershift EMSA with indicated antibodies. Lane 1 contains extract, but no antibody. Each antibody was tested in super shift assays at least 3 times.

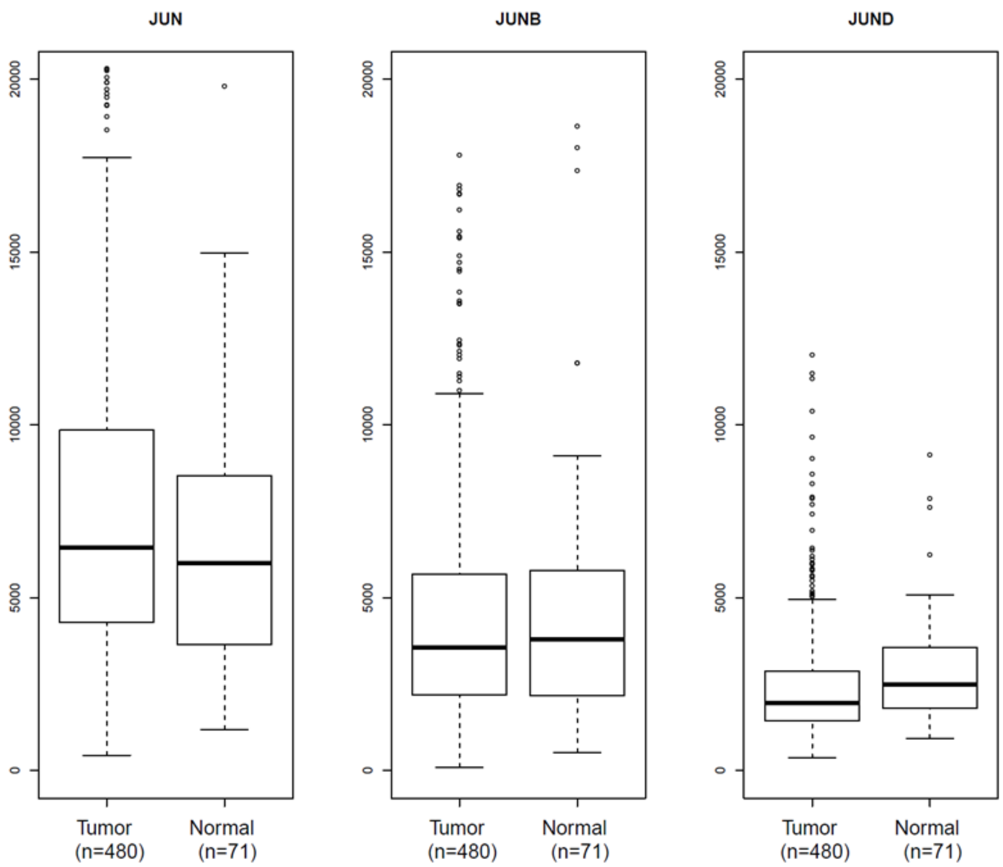

Supplementary Figure 3. Expression of (**a**) *JUN*, *JUNB*, and *JUND* in 71 normal renal and 481 tumor renal tissues from the TCGA database. The expression is shown as normalized read counts. Thick black lines in the center of boxes represent the median, box boundaries designate the 25th and 75th percentiles and whiskers extend to the minimum of either the data range or 1.5 times the interquartile range. Statistical outliers are plotted as points extending beyond the whiskers.

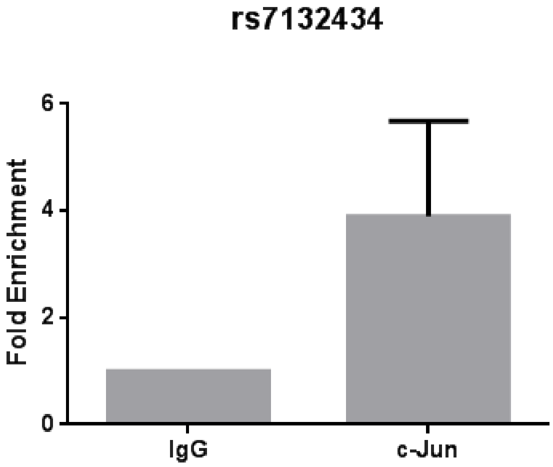

Supplementary Figure 4. ChIP with anti c-Jun (AbCam ab31419). Average fold enrichment relative to IgG for 5 independent experiments is plotted. qPCR assays were conducted in triplicate. Error bar represents the standard deviation. P = 0.02, Student's t-test, with two-tailed distribution.

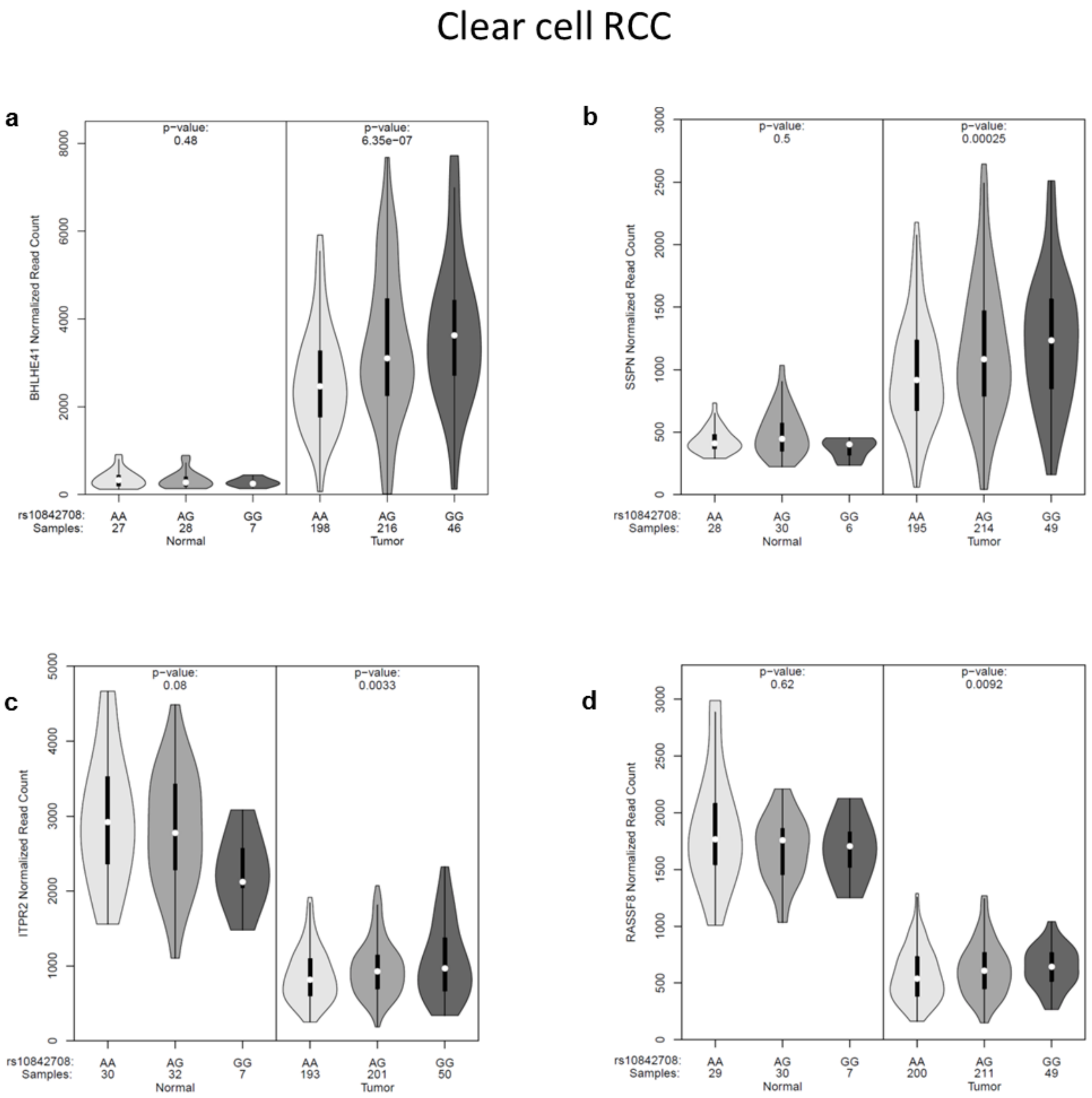

Supplementary Figure 5. eQTL analysis of RCC susceptibility variants on 12p12.1 in Clear Cell RCC. For the violin plots, the white dot represents the median, thick black line is the interquartile range (25th to 75th percentiles) and the whiskers represent the data range. P-values from a linear trend test. (**a**) The RCC risk allele rs10842708 (G) is associated with over expression of *BHLHE41* in clear cell renal carcinoma tissue (P = $6.35x10^{-7}$). The expression is shown as normalized read counts from 71 normal and 481 tumor renal tissues. Expression of (**b**) *SSPN*, (**c**) *ITPR2*, and (**d**) *RASSF8* for three genotypes of rs10842708.

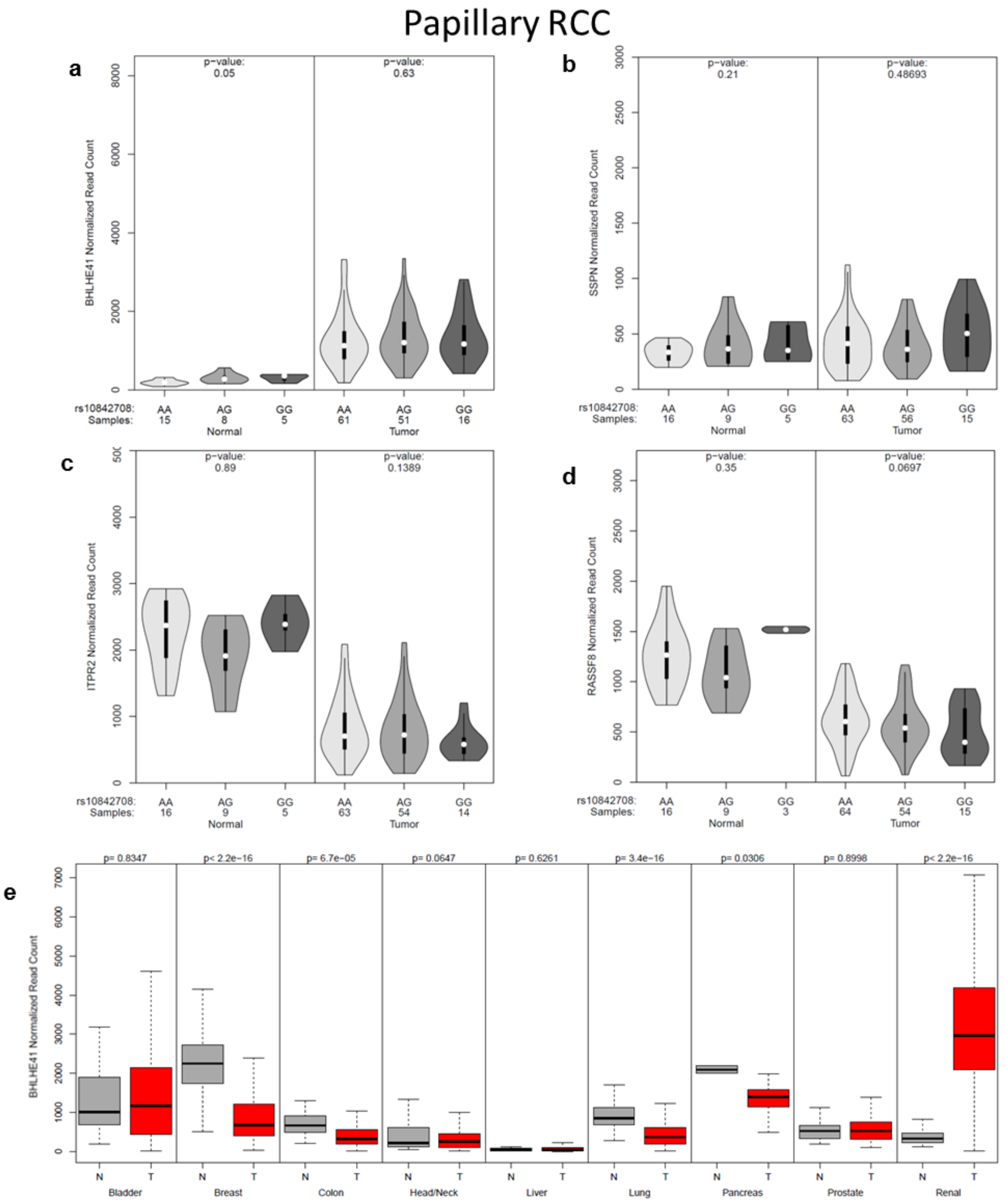

Supplementary Figure 6. eQTL analysis of RCC susceptibility variants in Papillary Renal Carcinoma. The RCC risk allele rs10842708 (G) is not associated with expression of (**a**) *BHLHE41*, (**b**) *SSPN*, (**c**) *ITPR2*, or (**d**) *RASSF8*. The expression is shown as normalized read counts from 30 normal and 141 tumor renal tissues. (**e**) Expression of *BHLHE41* in several tissues from TCGA. N, normal, grey; T tumor, red. In the violin plots (a-d), the white point represents the median, thick black line is the interquartile range (25th to 75th percentiles) and the whiskers represent the data range. In the boxplots (e), thick black lines in the center of boxes represent the median, box boundaries designate the 25th and 75th percentiles and whiskers extend to the data range. P-values are from linear trend tests.

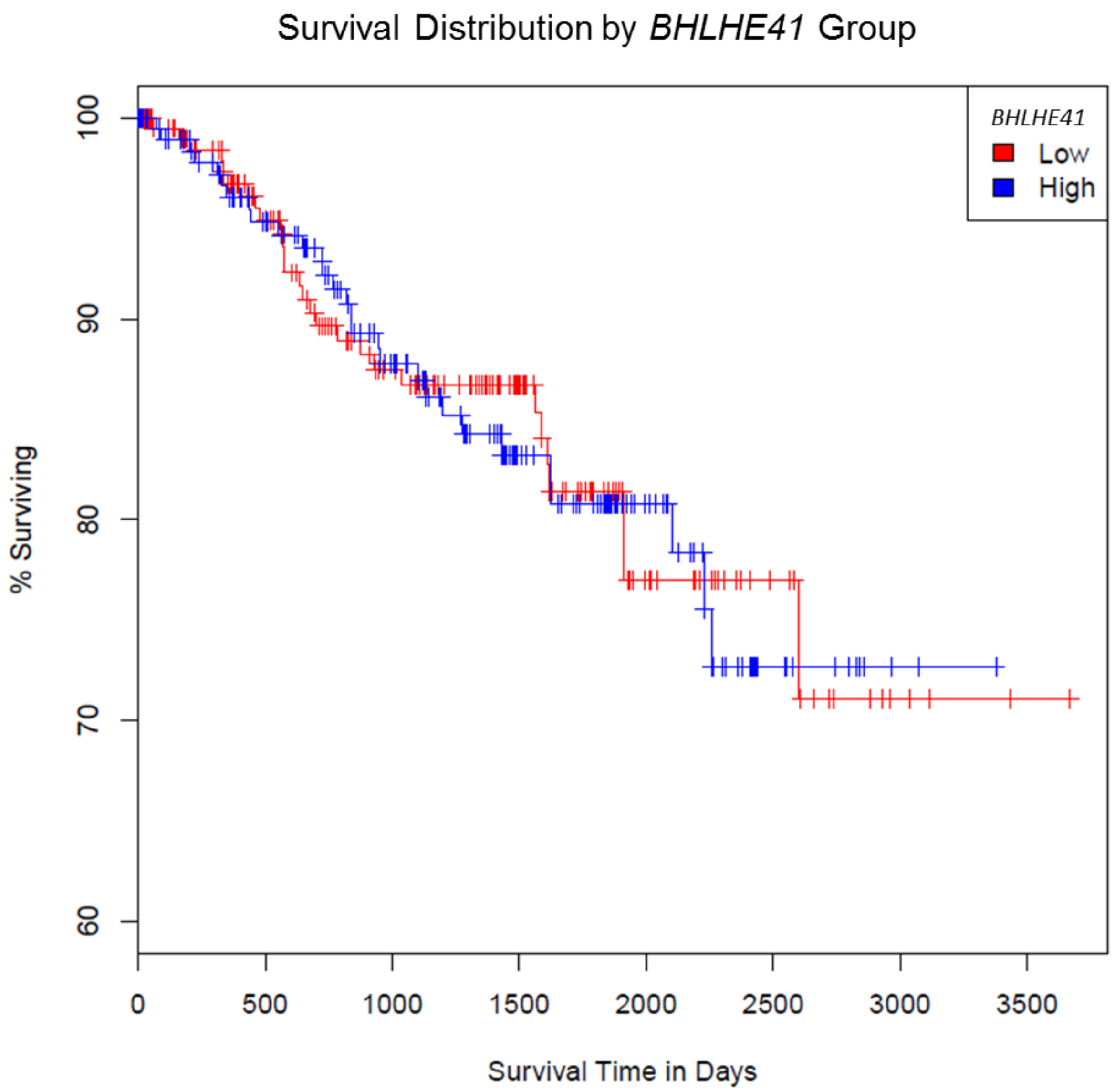

Supplementary Figure 7. Kaplan-Meier estimates of survival according to *BHLHE41* gene expression group (log-rank test = 0.924, see Methods for further details). Red, tumors expressing 'low' levels of *BHLHE41*; blue, tumors with 'high' expression levels.

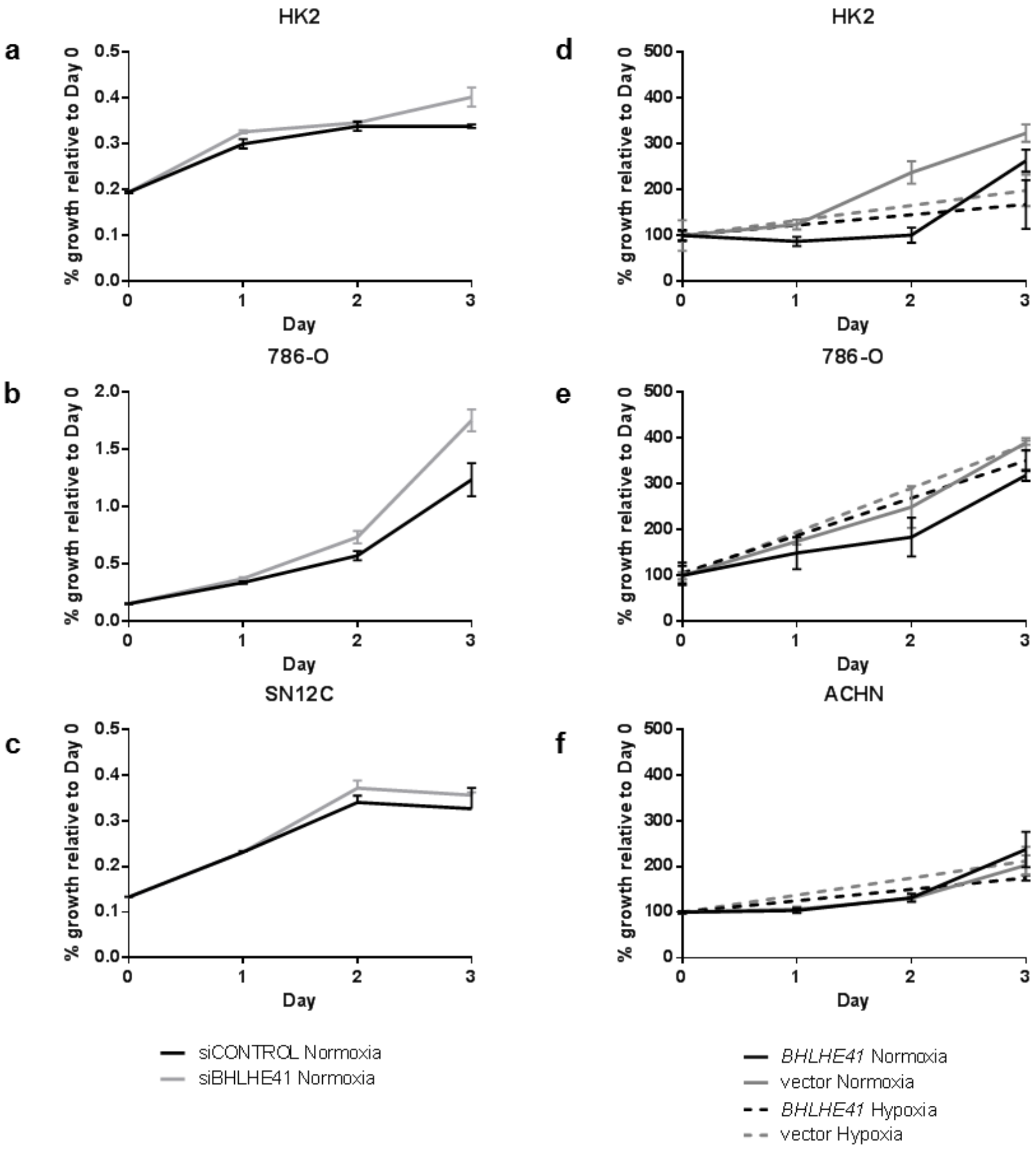

Supplementary Figure 8. Growth curves for (**a**) HK2, (**b**) 786-0, and (**c**) SN12C cell lines transiently transfected with siRNA. Cell proliferation for siRNA cells was measured using WST. N=3, error bars represent the standard deviation around the mean. Growth curves for (**d**) HK2, (**e**) 786-0, and (**f**) ACHN cell lines stably overexpressing *BHLHE41*, under normoxia and hypoxia. Cell proliferation assays for cells stably overexpression *BHLHE41* were measured using FluoReporter Blue Fluorometric dsDNA quantification kit (Thermo). N=3, independent experiments were performed in triplicate, error bars represent the standard deviation around the mean.

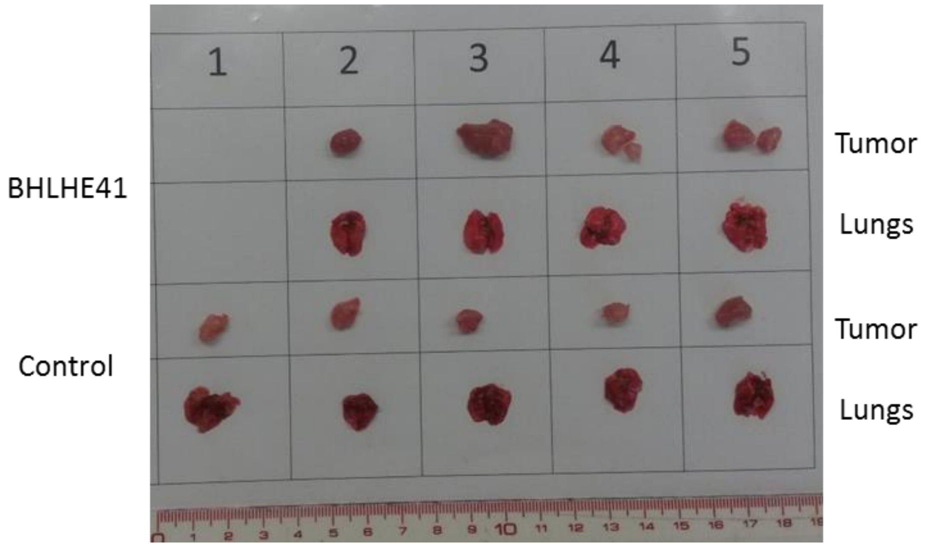

Supplementary Figure 9. Representative images of lungs and tumors harvested 6 weeks after xenograft with ACHN-*BHLHE41* or ACHN-vector cells. Ruler in cm is shown for scale.

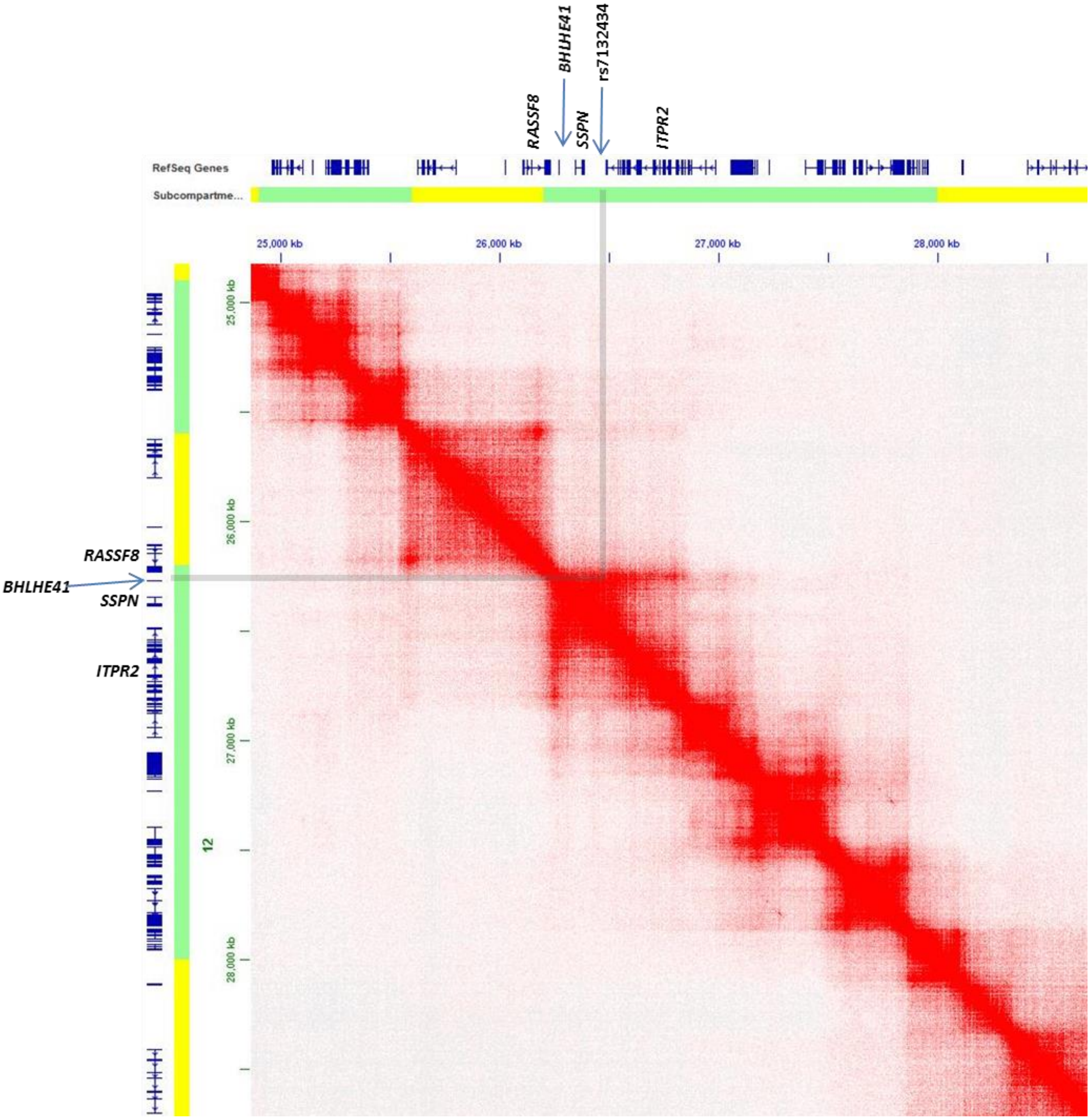

Supplementary Figure 10. Hi-C results from Rao and Huntley[1] for the 12p12.1 RCC risk region. Data can be found in GEO under the accession code GSE63525.

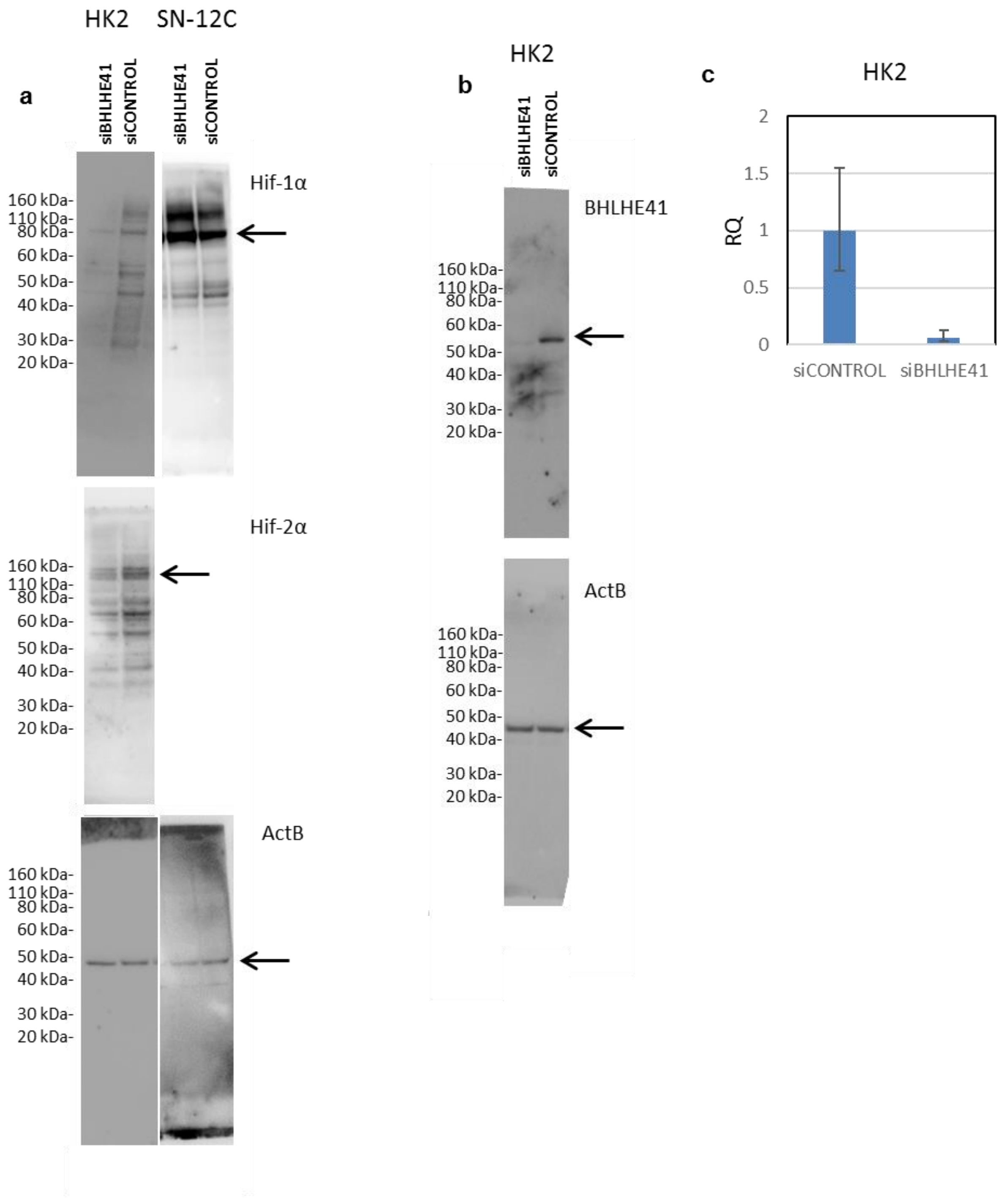

Supplementary Figure 11. Western blots. (**a**) Western blots of whole cell extracts of HK2 and SN-12C human renal cells transfected with siRNA and grown under hypoxic (1% O_2) conditions. Blots were probed with antibodies as indicated in the figure. (**b**) HK2 cells transfected with siRNA, and treated with 100 uM cobalt chloride for 24 hrs to mimic hypoxic conditions. Blots were probed first with antibody to BHLHE41, then stripped and re-probed for ActB. (**c**) qPCR for *BHLHE41* expression in HK2 cells 48 hrs after transfection with siRNA.

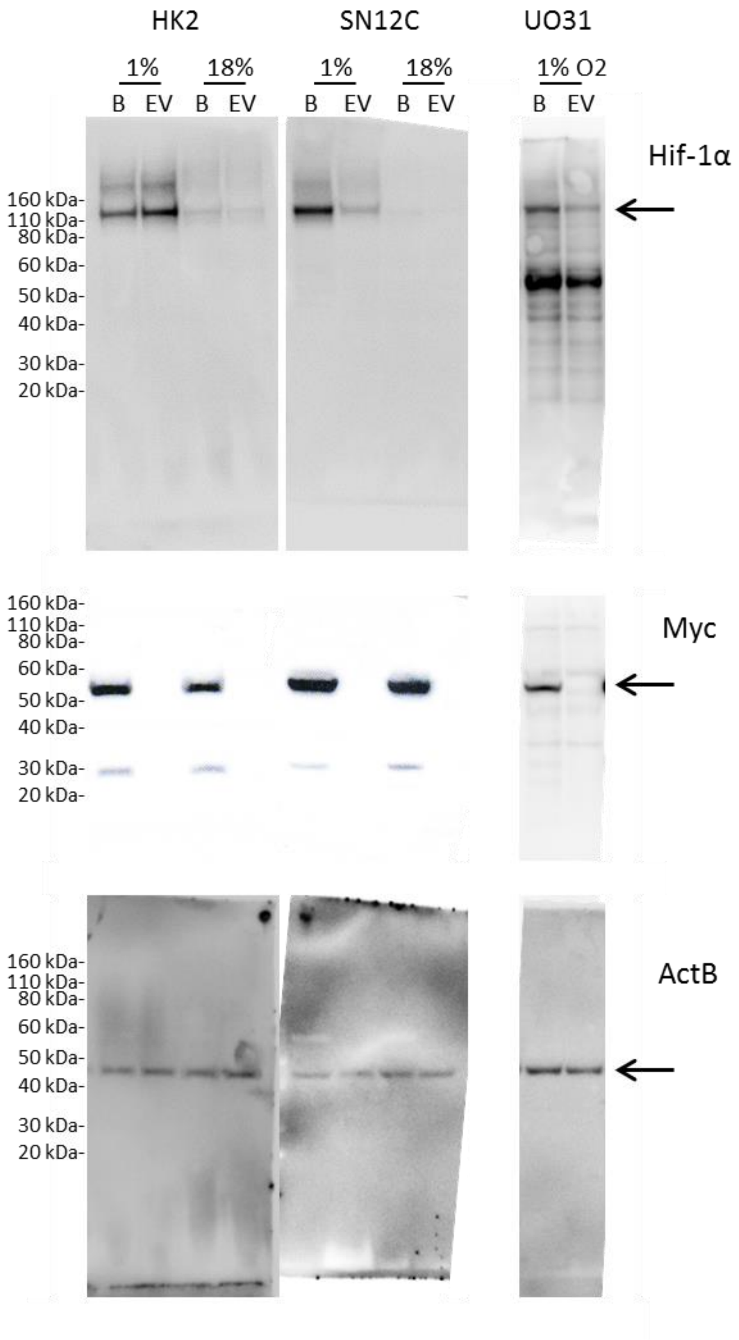

Supplementary Figure 12. Western blots of whole cell extracts of HK2, SN-12C, and UO31 cells 48 hrs after being transiently transfected with pcDNA3.1-*BHLHE41*-myc (B) or pcDNA3.1 vector (EV) under hypoxic (1% O_2) or normoxic (18% O_2) conditions.

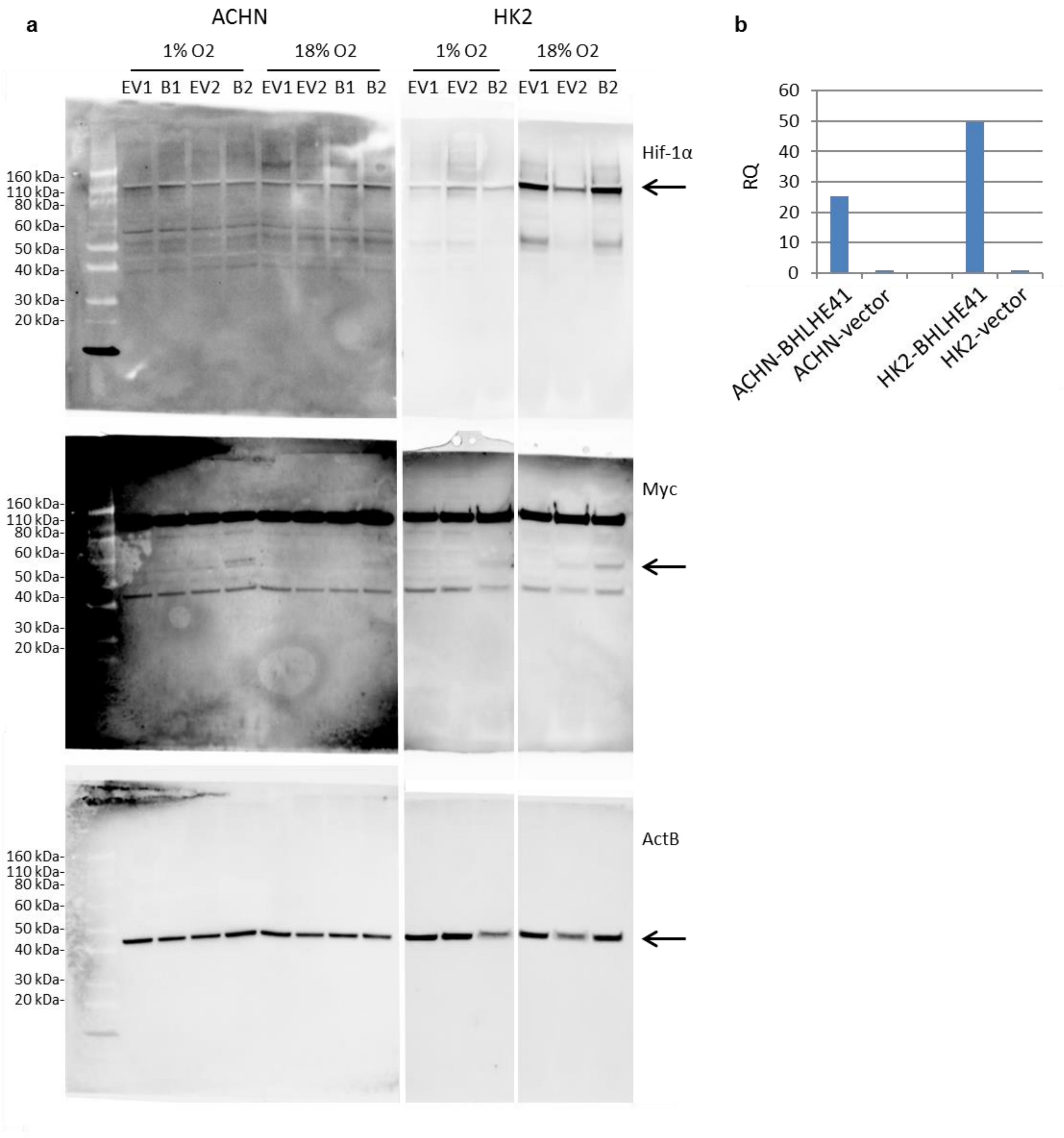

Supplementary Figure 13. Stable overexpression of BHLHE41 in renal cancer cell lines. (**a**) Western blots of whole cell extracts of HK2 and ACHN stably transfected with pCMV-*BHLHE41*-myc (B) or pCMV vector (EV) under hypoxic or normoxic conditions. Two isolates were selected for B and EV each. All western blots were repeated at least 3 times. (**b**) qPCR to measure *BHLHE41* expression, in HK2 and ACHN stably overexpressing *BHLHE41*.

Supplementary Table 1. SNPs demonstrating nominally significant associations with RCC risk ($p<5x10^{-4}$).

SNP	r^2 with rs718314	Genotyped	P-value
rs4963975	0.956	Yes[b,c]	2.13E-06
rs12814794	0.956	No	2.31E-06
rs718314	-	Yes[a,b,c]	3.44E-06
rs10842707	0.913	No	3.49E-06
rs10842705	0.913	No	3.92E-06
rs2129869	0.869	No	4.08E-06
rs7302344	0.64	No	4.24E-06
rs1049376	0.64	Yes[b,c]	5.00E-06
rs1027087	1	No	5.11E-06
rs1398676	1	No	5.22E-06
rs1049380	0.64	Yes[a,c]	5.27E-06
rs11048458	0.704	No	5.31E-06
rs4963979	0.956	No	5.49E-06
rs2137564	0.956	No	5.58E-06
rs1463679	1	No	5.59E-06
rs2175723	0.956	No	5.60E-06
rs10842704	0.956	No	5.63E-06
rs1872992	1	No	5.68E-06
rs10842703	1	No	5.68E-06
rs11048470	0.685	No	6.00E-06
rs10743579	0.956	No	6.04E-06
rs11048454	0.956	No	6.04E-06
rs10842708	1	No	6.30E-06
rs7132434	1	No	6.36E-06
rs11048456	0.957	No	6.43E-06
rs11534749	0.98	No	6.84E-06
rs4963661	0.99	No	8.09E-06
rs11048457	1	No	8.29E-06
rs12227542	0.98	No	9.07E-06
rs10842714	0.65	No	1.24E-05
rs11048447	0.719	Yes[c]	2.41E-05
rs10771276	0.47	No	2.72E-05
rs9442	0.507	Yes[a,b,c]	2.86E-05
rs4963983	0.54	No	3.11E-05
rs10842715	0.507	No	3.69E-05
rs4654	0.469	No	3.83E-05
rs2570	0.46	No	3.88E-05
rs10743582	0.48	No	3.89E-05
rs8311	0.441	No	3.93E-05
rs12829457	0.692	No	4.08E-05
rs10842702	0.402	No	4.83E-05
rs10842713	0.462	No	5.00E-05
rs7976877	0.507	No	5.47E-05
rs2048618	0.333	No	8.59E-05

(a) Directly genotyped in the International Agency for Research on Cancer (IARC) European ancestry samples (cases=1,936, controls=3,742)
(b) Directly genotyped in the Wellcome Trust Case Control Consortium (WTCCC) European ancestry samples (cases=350, controls=1,361)
(c) Directly genotyped in U.S. National Cancer Institute (NCI) European ancestry samples (cases=1,311, controls=3,424)

Supplementary Table 2 Genes showing differential expression in ACHN cells

gene	log2(fold_change)	gene	log2(fold_change)	gene	log2(fold_change)	gene	log2(fold_change)
CFHR2	inf	MAL2	-1.01829	PLIN2	-1.58253	CCL2	-2.4894
IRX6	inf	AMPD2	-1.03582	ANGPTL4	-1.58647	TCN2	-2.56915
IL11	4.7806	ELMSAN1	-1.05134	KCNQ1OT1	-1.5996	JMY	-2.57345
PLXNA2	4.21744	HKDC1	-1.05686	CEBPD	-1.63008	SMPDL3A	-2.60172
CPA4	4.15533	KANK2	-1.05893	OAF	-1.64197	CHSY3	-2.62552
FRMPD3	3.89708	VLDLR	-1.07566	CRISPLD2	-1.64384	PAQR5	-2.73719
KRT81	3.73926	PFKFB3	-1.10827	ADRB2	-1.66479	KCNQ3	-2.74741
EFEMP1	3.68384	COL6A1	-1.13268	ELF3	-1.68766	SORBS2	-2.76874
HNRNPLL	3.50043	DDIT4	-1.13448	SDPR	-1.7006	NPNT	-2.78843
GDNF	3.46511	VGF	-1.14778	C3	-1.72958	CD177	-2.81548
GFOD1	3.08665	ACSL1	-1.1627	ANK3	-1.73292	PSG3	-2.86082
PAK3	3.0425	TIMP2	-1.17702	NOV	-1.73568	ATP9A	-2.87343
NTN4	3.01499	PAM	-1.18508	CABLES1	-1.75045	THY1	-2.92018
IGDCC4	2.52002	SNTB1	-1.19556	PPL	-1.77356	TLR2	-2.92044
COL1A1	2.5146	EFHD2	-1.23215	KCNAB2	-1.78131	CLDN2	-3.2718
EMB	2.49581	PNRC1	-1.23238	DHRS13	-1.78435	PTCH1	-3.29396
GPC4	2.42869	SCD	-1.23957	MBP	-1.81326	ARHGAP24	-3.34405
BHLHE41	2.38012	BIRC3	-1.24171	CSF2	-1.86831	CSDC2	-3.38446
CHRDL1	2.23067	CXCL8	-1.2442	CNTNAP1	-1.90771	SMTNL2	-3.43163
BAAT	2.22841	GGA2	-1.27479	NDRG1	-1.94171	SLC16A7	-3.5009
C4orf26	2.21353	SEL1L3	-1.28648	HILPDA	-1.95677	PTGS1	-3.64958
KIAA1462	2.1595	PDGFB	-1.28747	COL12A1	-1.99011	ANKRD34B	-3.68729
WNT5B	1.99927	MYO1D	-1.32133	CDKN1C	-2.04422	SLC2A2	-3.8387
ADAMTSL1	1.97399	CXCL1	-1.32354	ADAMTS15	-2.05426	CHST9	-3.84866
ACAA2	1.90833	AMACR	-1.32426	MXI1	-2.09038	VCAM1	-4.06735
SCARA3	1.77145	FGFR1	-1.34635	CA12	-2.13511	COLEC10	-4.1676
ANKRD1	1.55499	LAMB1	-1.36025	ADM	-2.17796	NCALD	-4.24346
STOM	1.5256	TSPAN2	-1.39818	AGPAT9	-2.24643	TM4SF4	-4.33786
CHMP1B2P	1.52126	S1PR1	-1.42913	EDN2	-2.24892	NPR1	-4.61466
E2F7	1.4298	ARRDC3	-1.43523	SCARF1	-2.2989	MAL	-4.66062
SLC16A12	1.3974	GABARAPL1	-1.46527	DEPTOR	-2.36817	KCNJ15	-5.08927
SERPINE1	1.39514	FXYD2	-1.51144	GUCY1A2	-2.372	FOXQ1	-5.15047
TMCC3	1.314	IGFBP3	-1.5631	LAMA1	-2.37602	CFI	-5.17501
PRPS1	1.18601	FOXN3	-1.56391	PIK3IP1	-2.4208	PSG8	-5.27921
GPC1	1.12523	SPNS2	-1.57525	SECTM1	-2.44739	PRUNE2	-5.50978
				TNFSF10	-2.46717	SRGN	-6.86143

Supplementary Table 3. Oligonucleotides used in this study

Cloning	
Region 1:	
cloning -forward	TCAGCTCAGCAGTTATGTCTTTG
cloning -reverse	TGCATGATTCCCAGGTGTTA
Region 2:	
cloning –forward	CAGTTCACTGTTGAGAACTGCAC
cloning –reverse	TCAGCTGATCACACTGTCCAC
Region 3:	
cloning –forward	TGAGGCAGATGGAGATTTGA
cloning –forward	GCTGGAATGTGTCCACTCCT
EMSA	(only the forward strand is given)
rs7302344	
Minor allele	ATGTCTTGCTGTGTAAAATAG
Common allele	CTATTTTACATAGCAAGACAT
rs10842702	
Minor allele	ATAATGCCTTCAGGGTGCCCA
Common allele	ATAATGCCTTGAGGGTGCCCA
rs1027087	
Minor allele	TGGCAAAAGAGACAGGAGTCAT
Common allele	TGGCAAAAGAGTCAGGAGTCATT
rs2137564	
Minor allele	GGGCCTGGCAAGCAGTCTGAA
Common allele	GGGCCTGGCAGGCAGTCTGAA
rs7132434:	
Minor allele	ACAGTGACTCATCTGTGAGCT
Common allele	ACAGTGACTCGTCTGTGAGCT
rs10842707:	
Minor allele	GTCTTTGAATTTGAGTCTCAG
Common allele	CTGAGACTCAAATTCAAAGAC
rs718314:	
Minor allele	CCATTTGTCAGTCTGTCTGTC
Common allele	CCATTTGTCAATCTGTCTGTC
rs17383134:	
Minor allele	TTGGTAGTAGCTATTTCTGTG
Common allele	TGGTAGTAGTTATTTCTGTG
rs12814794:	
Minor allele	CTGTAGCGACGTGCAAGGGGT
Common allele	CTGTAGCGACATGCAAGGGGT
NFkB consensus oligonucleotide (sc-2505)	AGTTGAGGGGACTTTCCCAGG C
Stat3 consensus oligonucleotide (sc-2571)	GATCCTTCTGGGAATTCCTAGATC
Myc-Max consensus oligonucleotide (sc-2509)	GGAAGCAGACCACGTGGTCTGCTTCC
AP-1 consensus oligonucleotide (sc-2501)	CGCTTGATGACTCAGCCGGAA
ChIP	
rs7132434_ChIP_forward	TAACTTGGCCTGTCTGTTGC
rs7132434_ChIP_reverse	AATTGCTCAAGCCTCAGCTC

Supplementary Reference

1. Rao, S.S. *et al.* A 3D map of the human genome at kilobase resolution reveals principles of chromatin looping. *Cell* **159**, 1665-80 (2014).
